# Administrative Data Research Northern Ireland (ADR NI)

**DOI:** 10.23889/ijpds.v4i2.1148

**Published:** 2020-02-25

**Authors:** D O’Reilly, O Bateson, G McGreevy, C Snoddy, T Power

**Affiliations:** 1 Queen’s University Belfast; 2 Northern Ireland Statistics and Research Agency

## Abstract

**Background:**

The Administrative Data Research Northern Ireland (ADR NI), is a partnership between academia and the local statistics agency to advance the access to and use of administrative data in Northern Ireland. These goals are currently being advanced by undertaking a series of demonstrator Strategic Impact Projects developed to provide input to departmental areas of research interest and the current draft Programme for Government.

**Approach:**

ADR NI does not currently operate as a data repository but will negotiate access to and link subsets of administrative data from other departments and agencies as required for specific and approved projects. It is, however, anticipated that this model will broaden with the creation and retention of large linked datasets that could be used to address questions across a range of policy areas. At present accredited researchers can access the anonymised data only from within the safe setting situated in Belfast, although consideration is being given to using the Office for National Statistics Secure Researcher Service to access data more widely within the UK. ADR NI is currently being used to inform policy in a wide array of areas including health, education, environmental and urban planning.

**Discussion:**

ADR NI continues to develop and change. The growing confidence amongst data owners which has been supported by new and facilitating UK legislation has increased the willingness and ability to share and link administrative data. However, the associated logistical and administrative processes for accessing data will need further streamlining so that the timelines become more efficient and predictable. The evidence for the potential utility of research based on administrative data to influence policy continues to grow.

**Conclusion:**

Over the last decade we have demonstrated to data owners and the different publics that it is safe and acceptable to link administrative data for public benefit. The evolution and maturation of the ADR NI progresses apace and we continue to learn from developments in our sister organisations throughout the UK and throughout the world. We look forward to greater access to and use of administrative data both within Northern Ireland and across the UK.

## Background

The Administrative Data Research Northern Ireland (ADR NI) represents a partnership between academia (Queen’s University Belfast and University of Ulster) and the Northern Ireland Statistics and Research Agency (NISRA) – which is responsible for the collection and publication of statistics related to the economy, population and society of Northern Ireland: with a population of circa 1.9m. ADR NI is funded primarily by the Economic and Social Research Council, part of UK Research and Innovation (https://www.ukri.org/), with additional support from the Health and Social Care Research and Development Division (www.research.hscni.net). Along with ADR Scotland and ADR Wales, it is one of three national partnerships in the UK coordinated by a UK-wide Strategic Hub which also includes a partnership with the Office for National Statistics (ONS). The overarching aims of this Administrative Data Research Partnership are to facilitate safe and secure access for accredited researchers to administrative data in the UK predominantly in order to help inform and evaluate government policy decisions. This goal is currently being addressed primarily through a series of Strategic Impact Programmes which are specifically developed to reflect and provide input to departmental areas of research interest.

ADR NI has been in existence for six years, though was previously known as the Administrative Data Research Centre for Northern Ireland (ADRC-NI). However, it has its origins in the Northern Ireland Longitudinal Study (NILS)[1], which started in 1996. This is an ongoing large-scale, representative data-linkage study created by linking data from the Health Card Registration system (which is required for eligibility to the health service) to the decennial Census returns starting at 1981 and to administrative data for example vital events such as births, deaths and marriages registered with the General Register Office and migration events data (derived from Health Card Registrations). One of the major innovations and one that has differentiated NILS from its sister Longitudinal Studies is the potential to further link Heath and Social care data in what are called ‘distinct linkage projects’. NILS is thus a 30 plus year longitudinal data initiative for health related research, covering approximately 28% of the Northern Ireland population (approximately 500,000 individuals and accounting for approximately 50% of households in Northern Ireland).

The remit of ADR NI is to provide a secure and accredited research environment to enable agreed, ethically approved and world-class research to be conducted using administrative datasets that are routinely collected and analysed by Government departments and other bodies. The ultimate aims of the joined up approach are that the research questions are of manifest benefit to society and should inform the development and monitoring of public policy and help ensure that decision making is evidence based.

The aim of this short paper is to describe the current operating model and governance structures of the ADR NI, along with the types of data that are available, how they are linked and processed, and how researchers may access the data.

## Operating model

ADR NI does not currently hold or retain any data and no identifiable or indexed data are warehoused in NISRA, though this policy may change in future. Instead, it receives specified data subsets from data providers on a project-by-project basis for the creation of research datasets. The personal data are held temporarily while the linkage is being created. The research datasets are retained for the duration of the project as agreed by the data providers. On completion of a project, the data are either archived or destroyed in accordance to agreement from the data providers. Data can only be accessed in the NISRA secure environment (see below).

## Architecture and information technology

The ADR NI operates two separate networks both housed within NISRA headquarters building (Colby House in Belfast); one for the Trusted Third Party (TTP) functions and one for the Research Support Unit (RSU) functions; they are also administered under separate governance and management arrangements. The TTP network uses a dedicated secure network and access is limited to staff with sufficient security clearance to have access to personal identifiable data required for the matching process. Data are received via Secure File Transfer Protocol (SFTP), imported into this network using an approved encrypted USB device and once matching is complete the linkage keys are transferred onwards to the RSU network, again using an approved encrypted USB device. Upon successful transfer of the linkage keys, all personal identifiable data relating to a project are deleted. Consequently, no personal data or linkage keys are stored on the secure network. The RSU Network is an accredited closed network, known as the Research Network on which only de-identified data are stored. Staff and researchers access these data from dedicated PCs within the building, which are connected through a dedicated LAN. 

## Governance, legislation and management

NISRA hosts the Secure Environment where accredited researchers access data for ADR NI research projects. This operates under the 'Five Safes' principles which have been endorsed by the Information Commissioner’s Office (https://ico.org.uk/about-the-ico/who-we-are/northern-ireland-office/) and provide assurance for data owners, researchers and the wider public. **Safe projects**: projects are only approved when the following conditions are met; that data providers provide a letter of support for the project; that the project has a clear public benefit and has been approved by an official independent research governance approval panel; that there is an appropriate legal gateway for processing the data and that ethical approval is obtained and researchers commit to publish their results. **Safe data** are ensured by having a complete separation of TTP and RSU functions within NISRA so that no one person can see both personal and attribute data. TTP and RSU also have separate line management structures to Director level within NISRA, IT networks and physical structures. In addition, researchers access only bespoke extracts of data that have been approved through an approval panel and are subject to extensive disclosure assessments. **Safe people** means that all researchers must become Approved Researchers, have completed a mandatory training course and passed the associated assessment on the Five Safes and disclosure, have signed an agreement promising to protect the confidentiality of data and have an institutional guarantor. With regard to the **safe setting**, researchers can access the data only through the Secure Environment which is a strictly controlled area governed by policies, protocols and procedures to ensure data confidentiality. Researchers within the secure environment are supervised at all times by NISRA RSU staff. **Safe outputs**: all text, tables and charts produced by researchers are checked by the support staff who have been trained in statistical disclosure control to ensure safe outputs i.e. that all outputs are within the designated confidentiality constraints. Under no circumstances will individual level information leave the secure environment. All data to be released from the secure environment must comply with the RSU Disclosure Control Policy and the RSU Licence Agreement.

Sharing of personal data is legally allowable under Article 6 of the General Data Protection Regulation (GDPR) where there is an exemption for processing of personal data for statistics and research purposes. The processing of special category data is necessary for archiving purposes in the public interest, scientific or historical research purposes or statistical purposes in accordance with Article 89(1) based on Union or Member State law. This shall be proportionate to the aim pursued, respect the essence of the right to data protection and provide for suitable and specific measures to safeguard the fundamental rights and the interests of the data subject under Article 9(2) (j) of the GDPR. The Digital Economy Act (DEA, 2017) [2] research clauses will be used to process the ADR NI projects.

### Consent model

The data used in ADR NI projects are obtained from administrative sources. Consent has not been directly received from the data subjects for ADR research projects as it has been assessed that such provision would involve disproportionate effort. This circumstance is covered under the GDPR [3] where the provision of such information proves impossible or would involve a disproportionate effort, in particular for processing for archiving purposes in the public interest, scientific or historical research purposes, subject to the conditions and safeguards (Article 89(1)). Use of the administrative data for research purposes is reflected in the NISRA privacy notice [4].

### Data matching

NISRA has been carrying out matching exercises between administrative sources for over ten years. They have built up a wealth of knowledge and experience in this area and developed data matching techniques and methodologies based on the range of data made available to them. As most administrative data systems do not have common unique identifiers, records have to be matched based on the available information (usually name, address and date of birth). NISRA TTP uses a ‘rule based’ or deterministic data matching approach, referred to as match-keys.

However, as the recorded demographic information across datasets can occur in a number of different forms, a single match-key is insufficient and multiple match-keys are usually required and have been designed to resolve particular inconsistencies between match pairs. The highest level of matching is exact matching which links pairs of records that are identical on all matching fields. An example of a non-exact match-key is one constructed from the first two characters of an individual’s forename and surname (Bi-grams), combined with their date of birth and postcode district. Typically, the match-keys are processed in a stepwise manner starting with the most exact match-key and cascading through different variations of the available demographic information. Records are only linked on a match-key if it is unique on both datasets (i.e. one-to-one match). If multiple records match on a particular match-key then the link is not made and candidates are passed on as a residual to the next match-key. An example of the use of match-keys can be found in the online document ‘Data matching using Northern Ireland administrative data: a worked example’ [5].

This process ensures that matching work of the TTP is flexible and adaptable to varying data nuances, utilises all available information, ensures a high degree of coverage and accuracy, is repeatable, and can be automated and completed in a timely manner. With each ADRNI project, a small random sample of data matches are clerically checked to assess matching accuracy. The results of this exercise are reported in the standard TTP matching report.

The NISRA match-key methodology has been developed using a variety of data sources though mostly relating to work on Census Administrative Data (CAD, formerly Beyond 2011 project). Testing was carried out comparing results to existing ‘gold standard’ links [6] between datasets and in general, the methodology produced comparable match rates to the existing links (usually 90+% [7]); in most cases the match-key generated links had a high level of compliance with the existing links (99.5+%).

While the standard match-key methodology suffices for most projects, other methodologies may be used to enhance matching further depending on the characteristics of the data to be matched. These include associative matching (for example when other associated person information is available in both datasets e.g. Census Other Household Occupants, GRO Births Mother, Father, Baby) or ordered longitudinal data matching when potential match subjects have available changing demographic information over time (for example periodic health card registration downloads, maiden name, address changes).

The Linkage Diagram (Figure 1) illustrates how the linkage keys are generated by the TTP Linkage Service. The linkage keys are created for each project and deleted from the TTP network as soon as these have been successfully passed over to RSU. They are not updated at any stage, project re-linkage is treated as a new project and all processes are carried out again from beginning to end.

## Data sources

Table 1 shows the data, at the time of writing, that are currently available for accessing and linkage within the ADR NI processes. Work is ongoing to expand and add to this list; more contemporaneous information on data availability, along with the associated relevant metadata can be found on the NISRA RSU website [8]. It should be noted however, that although this is a record of the agreements ‘in principle’ to make data available for research teams, in practice, agreement to access particular subsets of individual datasets is determined on a project-by-project basis.

## Accessing administrative data in ADR NI: the user journey

There are three stages that the researchers and the data go through in the creation of the linked research dataset; the first is ensuring that the project is feasible, the second that it is formally approved and the third is the actual creation and checking of the datasets (see Figure 2). The first step starts when the research team makes an initial approach to the ADR NI support team. This usually entails a brief outline of what is being proposed, the datasets/variables requested (if known) and an indication of the levels of disaggregation required. This allows the researchers to be guided by appropriate support staff with the requisite topic-specific expertise. It is often useful at this stage to meet with the data providers to ensure that there is buy-in for the proposed research. At the end of this stage the support staff will complete a brief initial feasibility report and, if positive, progress to the next stage which is helping the research team through the approval process. All ADR projects require formal approval from the Approval Panel and a favourable opinion from a recognised ethics committee. The application to the Approval Panel (https://www.statisticsauthority.gov.uk/about-the-authority/better-useofdata-statistics-and-research/betterdataaccess-research/better-use-of-data/) which is submitted by the researchers is accompanied by a feasibility and privacy advisory assessment, supplied by the ADR NI support team. It is not unusual for the Approval Panel to seek further information before granting approval. The project approval triggers the third stage which is the creation and formal signing off of Data Sharing Agreements. This stage requires an exact specification of the variables required which can throw up other potential disclosure issues and may therefore be somewhat protracted. The support team also checks that all members of the research team are approved researchers and have undergone secure researcher training that is recognised by the UK Statistics Authority [9]. The data are brought together, using the TTP linkage mechanism and given a final disclosure check to minimise the risk of accidental identification within the research dataset. This can entail further discussions with the research team and aggregation or dropping of problematic variables. Researchers can only currently access data under direct supervision by RSU staff within the secure environment in Colby House. Consideration is being given to plans for remote access to these data for other researchers throughout the UK using the Office for National Statistics Secure Researcher Service on a project by project basis and with agreement from data providers.

## Exemplar case studies

 Below are three exemplar studies which show some of the current completed work. It is worth making the following points: firstly, the heavy reliance on health and health-related datasets and secondly, the ability to utilise the wealth of individual household and area-level data in the 2011 Census. The current accent on health is because of our long history of working with NILS and health related datasets and because health is often an antecedent or outcome of interest even for those without a primary interest in health. Recent projects have tended to move away from a health focus.

### Example 1: An exploration of the sociodemographic characteristics, educational attainment and self-reported health status of farmers in Northern Ireland.

The Department of Agriculture, Environment and Rural Affairs (DAERA) has a statutory duty under the 1998 Northern Ireland Act Section 75 to determine if new policies are likely to have a differential impact upon distinct societal groups, such as ethnic minorities, people with disabilities or of different religious persuasions. A previous evaluation of the NI farming community required a dedicated and expensive survey. In 2014, DAERA, in conjunction with academic colleagues undertook a study to link the results of the annual Agricultural Census and EU Farm Structure Survey (which collects information on farms, farm structure and activity (including crop areas, livestock numbers, diversification activities, renewable energy, and farm labour) to the 2011 Population Census. The rich Census data was used to provide valuable insight on the educational attainment and health of farmers, along with their socioeconomic characteristics and the impact of long working hours. DAERA estimate that this project provided much higher quality population-wide data sets than generated by the previous survey and saved £350,000 ($438,000) in primary data collection costs. The emerging research results (see [10]) will impact DAERA’s remit to provide equality of opportunity to training and programmes, as well as actions within the Rural Development Programme and the Targeting Rural Poverty and Social Isolation (TRPSI) programme.

### Example 2: An examination of the effects of aircraft noise on mental health.

The increasing demand for air transport means more and bigger planes, and extended runways and flight times, so that more residents are exposed to aircraft noise. While there is clear evidence that aircraft noise is associated with cardiovascular disease related incidence and mortality, relatively little is known about the long-term influences on mental health. The aim of this study was to link individual 2011 Census records and modelled noise contours surrounding George Best Belfast City Airport, using the Census data as both the outcome measure, in terms of self-reported chronic poor mental health, and to adjust for demographic, socio-economic status and comorbidity confounders. A geographical information system, using point-in-polygon techniques, was used to locate the georeferenced Census households within noise contour polygons; residents being assigned a noise level based on the contour into which the household fell. The analysis did not detect an independent association between aircraft noise and mental health after adjustment for socioeconomic status [11]. However, this may be because it is a relatively small airport with no night-time flights. There are plans to extend this approach to larger and busier UK airports and to incorporate medications data as proxy measure of both mental health and sleep disturbance. A similar GIS-based approach is currently being used (with the additions of prescription data) to study the effects of air pollution at the extremities of age.

### Example 3: Using data linkage to understand the needs for, and use of, mental health services of migrants in Northern Ireland.

Northern Ireland has until recently been a very homogenous society and our knowledge of possible shortfalls in the service response to the needs of migrants lags behind those of other parts of the UK. This study used the ADRC-NI to combine data from a centralised prescriptions database, held by the health service, to 2011 Census records. The aims were (i) to use the self-report from the Census records to compare the prevalence of poor mental health in the migrant and settled communities and (ii) to determine if migrants were less likely than the settled community to receive appropriate treatment for poor mental health by examining levels of prescribed medications over the following 12 months for those who reported chronic poor mental health. The data showed that although migrants generally report both good physical and mental health, there is evidence of underreporting of mental ill-health amongst some migrants groups, and that most migrant groups are much less likely to be in receipt of psychiatric or psychological medications [12,13]. The research team is undertaking parallel comparative analysis in Finland. The study has stimulated much debate and the aim is to bring all of the migrant-related record-linkage work together in a symposium comprising relevant stakeholders to determine what the subsequent policy and research responses should be.

## Discussion

Data availability is the lifeblood of administrative data research and although ADR NI has made significant advances over recent years resulting in getting agreement from data owners to provide an extensive portfolio of administrative data to be used for research, we continue to negotiate with other Government Departments and data owners to further extend this list and bring other datasets within reach. We are also seeking to enable the reuse of linked datasets so that they can, with the agreement of the respective data owners, be made available to other researchers to answer new questions. However, it should be acknowledged that there is limited scope for reuse of the datasets that have been generated to date as they have been very tightly defined and approved so as to address a limited research agenda. A more fruitful approach will be the ongoing construction of larger multipurpose datasets under a *‘create once-use many times’* approach. This will replace the extant *‘create and destroy’* principle and will be much less resource intensive for the data owners and provide more efficient vehicles for research. An example of these themed datasets is CASHE, a new linkage that ADR NI is creating between Annual Survey of Hours and Earnings (ASHE 2011, which examines work patterns and earnings across the private and public sectors) and the 2011 Census. The linked dataset will facilitate research about working life in NI and, because it aligns closely with a similar ONS linkage, will enable UK comparisons to be drawn.

ADR NI also aims to improve access to data to researchers across the entire UK. This means increasing access to data from other parts of the UK within the ADR NI safe setting, but also eventually making NI data accessible to researchers in Great Britain. The safe setting already hosts a dedicated terminal which is part of the ONS Secure Research Service (previously known as the Virtual Microdata Laboratory (VML) [14]) and that provides secure access for Approved Researchers to sensitive detailed data held by the Office for National Statistics (ONS). Such data can be accessed via, but not downloaded onto, these dummy terminals. There is also a separate terminal that facilitates access to data from Wales via the Secure Anonymised Information Linkage (SAIL) and we are actively exploring access to Scotland administrative data from within the safe setting.

Furthermore, it is clear that, to date, most of the analyses across the ADR UK have been by researchers within own jurisdictions. This is also true of the ADR NI, i.e. that most of research on administrative data in NI is done by NI researchers. However, the policy variations arising from devolution across the constituent parts of the UK have produced natural experiments that could be exploited to evaluate areas where there has been policy divergence. However, capitalising on these opportunities will require physical or statistical pooling of the data across the UK. Statistical techniques such as eDataShield [15] will provide a workable interim solution. This technique facilitates combined analysis of physically separated datasets, including datasets such as the Census where strict security access usually prevents these data being pooled [16,17]. However, there is obviously a potential need to move towards scenarios where the individual-level data across the UK are combined and analysed in one setting.

## Ethics statement

The study did not require ethical approval as it was a descriptive paper and not a research study.

Figures & Tables

**Figure 1: This is a diagrammatical representation of the steps by which data from two separate organisations within Northern Ireland are safely and securely linked using an independent, trusted third party linkage centre:**
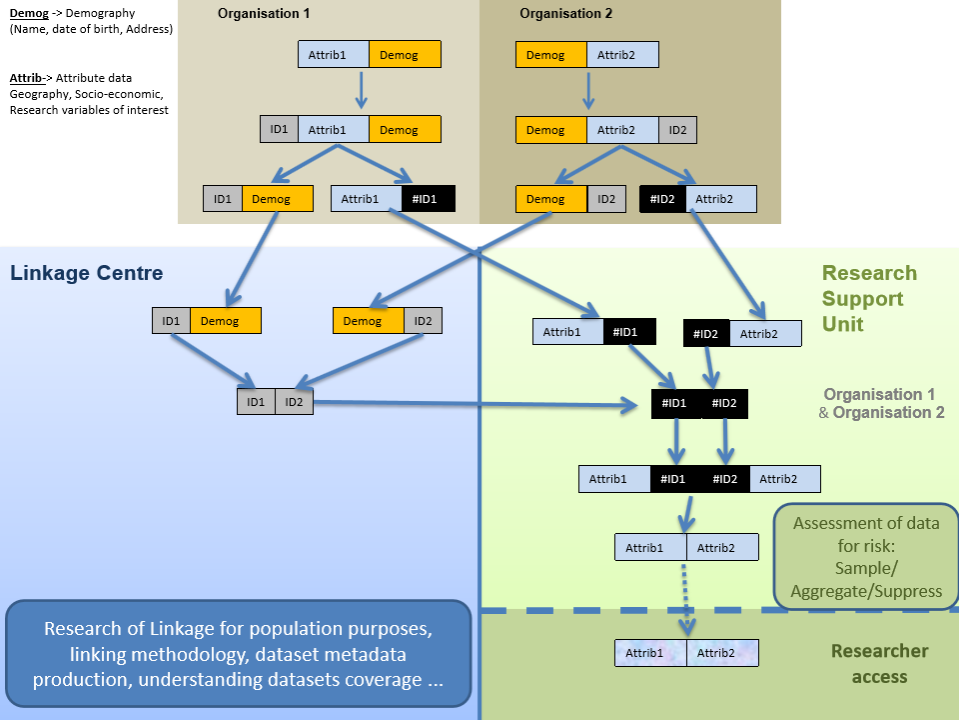


**Figure 2: These are the steps and stages that a typical Administrative Data Research (ADR) project will have to go through from the concept stage through to the production of a research-ready dataset for research use within the safe setting.**
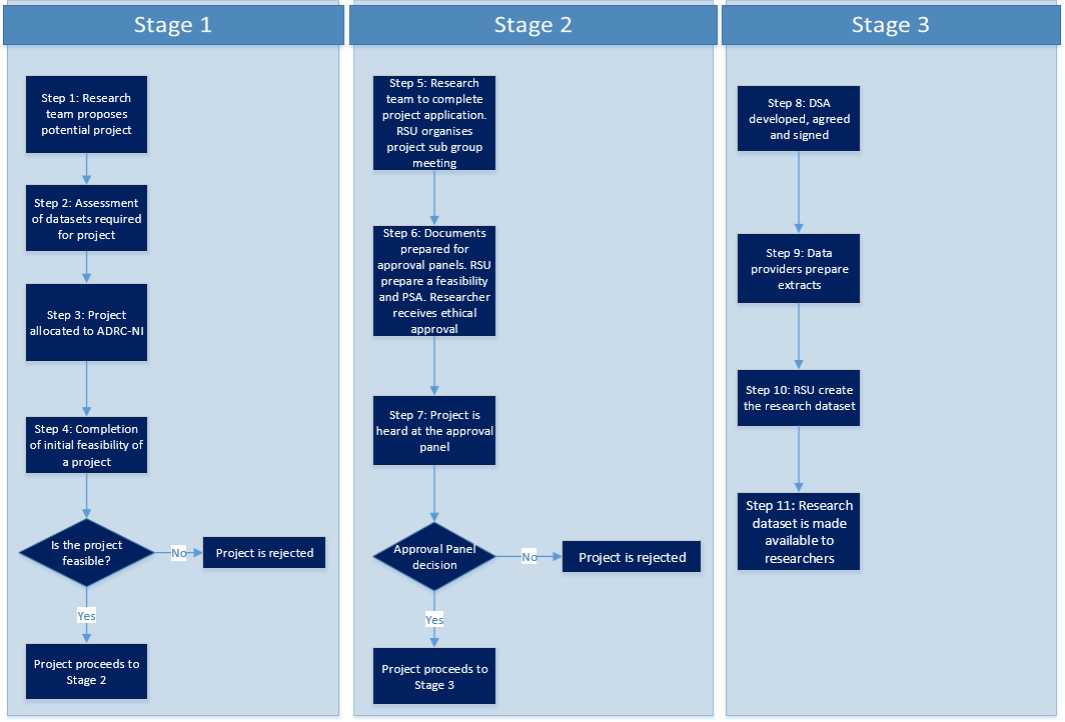


**Table 1: Schema of data sources currently available:** 

Data owner	Data	Years covered
Census office	Decennial population Census	2001 & 2011
General Register Office	Mortality records	1997-2013
	Birth records	1997-2013
Land and Property Service	Valuation List of Domestic and non-domestic properties	2008-2016
Department of Agriculture, Environment and Rural Affairs	Agricultural Census in Northern Ireland	2011-2016
	Air quality records	1986 to present
	Water quality	1986 to present
Electoral Office Northern Ireland	Electoral Register (roll)	2013-2016
Department for Communities	Social security benefits records	2011-2016
Department for Infrastructure	Planning activity	2011-2016
Department for the Economy	Higher Education enrolments & qualifications & destinations	Academic Years 2006/07 to 2015/16
Department of Education	School Census (enrolment records)	2010-2016
	School Leaver's Survey (qualifications and destination)	2010-2016
Business Services Organisation **	GP Register (NHAIS)	2010-2017
	Dental Statistics	2010-2017
	Pharmaceutical (EPD)	2010-2017
	Ophthalmic Statistics	2010-2017
